# Small Molecule Based Organic Photo Signal Receiver for High‐Speed Optical Wireless Communications

**DOI:** 10.1002/advs.202203715

**Published:** 2022-10-03

**Authors:** Seonghyeon Cho, Chul‐Joon Heo, Younhee Lim, Seoyeon Oh, Daiki Minami, Minseok Yu, Hyunchae Chun, Sungyoung Yun, Hwijoung Seo, Feifei Fang, Jeong‐Il Park, Cheol Ham, Jisoo Shin, Taejin Choi, Juhyung Lim, Hyeong‐Ju Kim, Hye Rim Hong, Hiromasa Shibuya, Jeoungin Yi, Byoungki Choi, Kyung‐Bae Park

**Affiliations:** ^1^ Dept. of Information and Telecommunication Engineering Incheon National University Incheon‐si 22012 Republic of Korea; ^2^ Organic Materials Laboratory Samsung Advanced Institute of Technology (SAIT) Samsung Electronics Co. Ltd. 130 Samsung‐ro, Yeongtong‐gu Suwon‐si Gyeonggi‐do 443‐803 Republic of Korea; ^3^ CSE team Innovation Center Samsung Electronics Co. Ltd. 1 Samsungjeonja‐ro Hwasung‐si Gyeonggi‐do 18448 Republic of Korea; ^4^ Energy Excellence & Smart City Lab., Incheon National University Incheon‐si 22012 Republic of Korea

**Keywords:** bulk heterojunctions, laser diodes, machine learning, optical wireless communications, organic photodiodes, visible light communications

## Abstract

The present work describes the development of an organic photodiode (OPD) receiver for high‐speed optical wireless communication. To determine the optimal communication design, two different types of photoelectric conversion layers, bulk heterojunction (BHJ) and planar heterojunction (PHJ), are compared. The BHJ‐OPD device has a −3 dB bandwidth of 0.65 MHz (at zero bias) and a maximum of 1.4 MHz (at −4 V bias). A 150 Mbps single‐channel visible light communication (VLC) data rate using this device by combining preequalization and machine learning (ML)‐based digital signal processing (DSP) is demonstrated. To the best of the authors' knowledge, this is the highest data rate ever achieved on an OPD‐based VLC system by a factor of 40 over the previous fastest reported. Additionally, the proposed OPD receiver achieves orders of magnitude higher spectral efficiency than the previously reported organic photovoltaic (OPV)‐based receivers.

## Graphical abstract

Organic photodiodes (OPDs) have high potential in industrial and commercial applications for their unique characteristics. The development of a small molecule‐based organic photo signal receiver for high‐speed optical wireless communications is described. The optimized bulk heterojunction‐OPD receiver with the proposed communication scheme combining device‐oriented preequalization scheme and machine‐learning technique leads to a fast and power‐efficient OPD‐based optical wireless communication system.

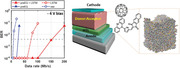

## 1 Introduction

Radio frequency (RF)‐based internet‐of‐things (IoT) devices and sensors have grown exponentially in recent decades. These include smart devices powered by artificial intelligence (AI), autonomous vehicles, virtual reality (VR), augmented reality (AR), and mixed reality (MR). As a result, the exponential growth of data traffic has led to an unavoidable bandwidth crunch. This ever‐increasing demand for RF spectrum resources has fueled interest in the use of spectrum other than RF for wireless communication.^[^ ^1^, ^2^ ^]^

Recently, optical wireless communication (OWC) has gained attention as a promising solution for alleviating the bandwidth crunch.^[^ ^3^, ^4^ ^]^ It has an unlicensed and unlimited optical spectrum that spans a much wider frequency band than its radio frequency counterpart. OWC technologies, which include visible light communication (VLC), light fidelity (LiFi), and free‐space optics (FSO), enable advanced wireless communication with high data transmission speeds, high security, and low energy consumption.^[^ ^1^, ^5^ ^]^

Typically, VLC systems detect modulated optical signals carrying a variety of data using silicon‐based photodiodes (PD), such as positive‐intrinsic‐negative (PIN) PD and avalanche PD (APD). However, the detection area of these receivers is typically limited, resulting in low‐power reception.^[^ ^6^ ^]^ This can be compensated for by using a compound parabolic concentrator (CPC) or a fluorescent concentrator (FC)^[^ ^7^ ^]^ to concentrate light into the detection area. However, it frequently necessitates a precise beam alignment technique between the transmitter and receiver, or it often requires sophisticated optimization of fluorescent materials and optomechanical designs. Additionally, inorganic PDs require an external bias voltage to operate. A higher external bias voltage is required, in particular, for an avalanche operation utilizing APDs.

Organic photodetectors, such as organic photodiodes (OPD) and organic photovoltaics (OPV), can be manufactured with a large detection area and a high optical gain. Additionally, these can be operated without an external bias voltage, extending their application range. The optional external bias voltage results in increased efficiency and response time.^[^ ^8^, ^9^, ^10^ ^]^ Moreover, the lightweight organic photodetector can be fabricated on thin and flexible substrates. Reference^[^ ^11^ ^]^ demonstrated the flexible OPD's industrial possibilities. Through cost‐effective printing techniques, low‐temperature processes, and coating techniques, it enables mass production of wearable and compact IoT devices.^[^ ^12^, ^13^, ^14^ ^]^ Additionally, indoor OWC with light sources such as light emitting diode (LED) or laser diode (LD) with organic detectors have been researched.^[^ ^15^, ^16^ ^]^

As a result of these considerations, the use of organic photodetectors as OWC receivers has garnered attention. Utilizing DC‐biased optical orthogonal frequency division multiplexing (DCO‐OFDM), OPV‐based VLC systems with energy harvesting and data communication have been demonstrated.^[^ ^17^, ^18^ ^]^ Reference^[^ ^18^ ^]^ recently introduced a 4‐by‐4 multiple‐input multiple‐output (MIMO) structure based on OPV, as well as a single‐channel structure, with data rates of 363 Mbps and 147.5 Mbps, respectively.

Especially, OPD can be designed to detect a specific wavelength range without the use of additional filters, which can be applied to high‐resolution full‐color organic image sensors.^[^ ^14^ ^]^ Additionally, its superior performance has been reported, including a low dark current density of 0.11 nA cm^−2^, a high responsivity of 0.4 A W^−1^, and a detectivity of up to 9.2 × 10^12^ Jones.^[^ ^19^ ^]^ Also, OPD may have a simpler structure that enables widespread deployment in practical applications. For these practical advantages, the potential of OPD‐based receivers has been researched worldwide.

Reference^[^ ^20^ ^]^ demonstrated an OPD‐based VLC system with an equalizer to reduce inter‐symbol interference (ISI). The authors demonstrated a data rate of 3.75 Mbps using an artificial neural network (ANN) equalizer and non‐return‐to‐zero on‐off keying (NRZ‐OOK). In an OPD‐based VLC system, reference^[^ ^6^ ^]^ used a predistortion scheme. It used pulse amplitude modulation (PAM) to achieve the 125 kbps data rate with a 7% forward error correction (FEC) error rate. The OPD bandwidths and the ratio of the achieved data rate to the bandwidth are 160 kHz (23.4 bps Hz^−1^)^[^ ^20^ ^]^ and 8 kHz (15.6 bps/Hz)^[^ ^6^ ^]^ in these studies, respectively. Also, ANN‐based fully organic (organic LED and OPD) communication link achieving 1 Mbps from 135 kHz bandwidth was demonstrated.^[^ ^21^ ^]^

While these previous studies demonstrated the feasibility of OPDs as OWC receivers, the data rates reported thus far are significantly lower than those required for a variety of future wireless applications, including connected cars (>10 Mbps), AR/VR/XR (10–100 Mbps), and wireless cloud‐based offices (≈100 Mbps).^[^ ^22^ ^]^ The low bandwidth of OPD is regarded as a significant impediment to speeding up the OPD‐based OWC system. Thus, increasing the bandwidth of OPD devices is critical. Additionally, it is critical to develop the most appropriate communication methods that adequately compensate for the low bandwidth OPD‐based receivers.

The present work introduces a high‐bandwidth OPD device with a bandwidth of 1.4 MHz, which is nearly a factor of ten faster than the fastest previously reported. Additionally, much faster data rates are demonstrated through the convergence of a preequalization and machine learning (ML)‐based digital signal processing technique (DSP). The data transfer rates achieved are 30 Mbps (zero bias) and 150 Mbps (−4 V bias). To the best of the authors' knowledge, these are the fastest data rates ever achieved on an OPD‐based VLC system, with an improvement of x8 (zero bias) and x40 (−4 V bias) over the results published previously.

## 2 Results

### 2.1 OPD Structure and Characteristics

**Figure 1:**
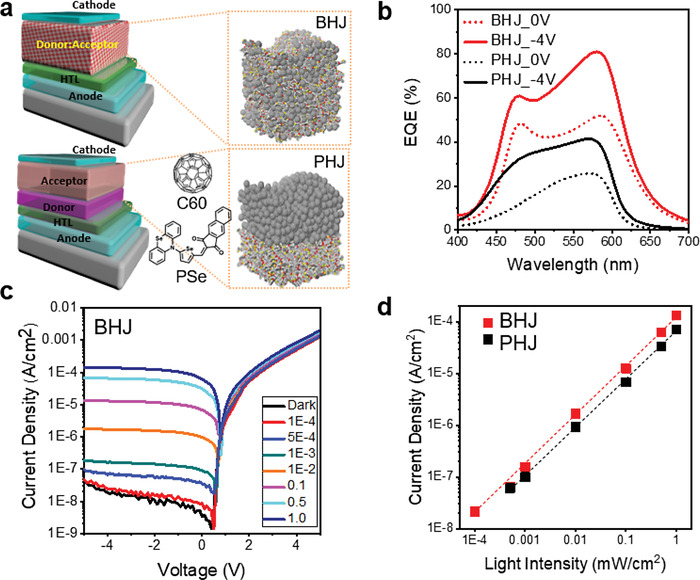
OPD structure and photon‐to‐electrical conversion characteristics. a) Schematic diagrams of two different types of OPD devices and their photoelectric conversion layers; BHJ and PHJ. b) EQE spectra of both devices under 0 V and −4 V. c) Photo *J–V* spectra of BHJ‐OPD varying intensity of incident light. d) Photocurrent density properties at an operating voltage of −4 V as a function of the light intensity.

By incorporating a pair of metal electrodes, we were able to fabricate OPD devices with a high conversion efficiency and rapid response speed. The device structure was optimized for use as a photo‐receiver for VLC. Narrow‐band OPDs can selectively absorb light with a high conversion efficiency and response speed, making them applicable to VLC.^[^ ^23^, ^24^ ^]^ Under fixed bias conditions, the photo‐response speed of OPD is strongly dependent on the effective electrical field. By incorporating a pair of metal electrodes onto OPDs, we were able to fabricate a thin OPD receiver capable of operating at MHz‐level response speed and bandwidth. Organic electronic devices can be manufactured using a variety of techniques, including vacuum thermal deposition, solution casting, and inkjet printing.^[^ ^25^, ^26^, ^27^ ^]^ We chose vacuum thermal deposition processes for the fabrication of OPD receivers because they are compatible with other silicon fabrication processes, and the possibility of large‐scale production has already been demonstrated in the field of OLEDs. As illustrated in **Figure** **1**a, the OPD was constructed on a reflective electrode substrate using a hole transfer layer (HTL), a photoelectric conversion layer, and a thin semi‐transparent electrode. By employing a pair of metal electrodes, the total thickness of OPD was reduced to less than 100 nm (80 nm), and the effective electrical field applied to OPD was maximized when an external electrical bias was fixed. A 30 nm thick HTL was deposited on a glass substrate with an ITO/Ag/ITO pattern to aid hole transfer from the photoelectric conversion film to the anode and to inhibit unwanted leakage current flow through the OPD. We prepared two distinct types of photoelectric conversion layers to determine the optimal design for VLC applications and compared their device performance: bulk heterojunction (BHJ) and planar heterojunction (PHJ) heterojunctions.^[^ ^28^, ^29^, ^30^ ^]^ As a communication receiver, the most critical properties of OPD are its rapid response to rapidly changing light signals and photon‐to‐electrical conversion efficiency. Charge separation and transfer characteristics vary significantly depending on the type of photoelectric conversion film used, despite the fact that both devices are composed of the same materials. The absorption layers were deposited using donor and acceptor small materials to create a well‐mixed junction (BHJ^[^ ^8^ ^]^) or a layered junction (PHJ^[^ ^31^ ^]^). As a donor material, a novel donor–*π*–acceptor molecule (PSe) was used, which consisted of two fused‐type heterocyclic donors and an electron‐accepting unit. As an acceptor, the well‐known fullerene was used.^[^ ^32^, ^33^ ^]^ To reduce the capacitance of the device, a semi‐transparent metal cathode (15 nm, Ag) was deposited on the top of the device. The semi‐transparent cathode developed in this study has a sheet resistance of 15.4 ohm sq^−1^ and a transparency of 67.8%. Incident light is absorbed in the photoelectric conversion layer, reflected from the anode, and partially reflected from the cathode again, and this phenomenon can be used to induce optical resonance and thus increase the absorption efficiency. Due to the optical resonance in OPD, incident light is absorbed maximally through the photoelectric conversion layer, and incident photons are effectively converted to electrons in each junction interface of the donor and acceptor molecules.^[^ ^34^, ^35^, ^36^ ^]^ By introducing a semi‐transparent top electrode and reflecting bottom electrode on OPD, the total thickness was reduced, and response speed could be improved without losing their sensitivity performance. (See Note S1, Supporting Information.) We investigated the fundamental properties of two different OPDs (BHJ, PHJ) by measuring g their external quantum efficiency (EQE; *η*), bias‐dependent current density properties under light illumination (photo *J–V*), and photocurrent linearity with varying light intensity [linear dynamic range (LDR)]. EQE was used to investigate the photoelectric conversion property. The EQE of the OPDs was calculated by multiplying three types of efficiencies, including absorption (*η*_A_), charge separation (*η*_cs_), and charge collection (*η*_cc_) as follows:

(1) $$\mathrm{EQE}\left(\%\right)=\eta_{A}\times \mathrm{IQE}=\eta_{A}\times \eta_{\mathrm{cs}}\times \eta_{\mathrm{cc}}$$

where IQE is internal quantum efficiency. The EQE of OPDs was investigated when applied at 0 V and −4 V (Figure 1b). At 550 nm, the EQE of BHJ‐OPD is 45.6% at 0 V and 72.7% at −4 V, which is sufficient for use as a VLC receiver for converting incident photons to electrical signals.^[^ ^14^ ^]^ In comparison to BHJ‐OPD, PHJ‐OPD exhibits a lower EQE of 24.5% and 39.9% at 0 V and −4 V, respectively. To investigate the photo *J–V* properties of BHJ‐OPD and PHJ‐OPD, photo *J–V* measurements were made and the results are shown in Figure 1c,d. The photo *J–V* spectra of BHJ‐OPD were determined and compared using a light intensity range of 1E‐4 to 1.0 mW cm^−2^. The linear dynamic range (LDR) characteristics of both types of OPDs were determined using the equation below.^[^ ^37^ ^]^

(2) $$\mathrm{LDR}=20\;\log\left(\frac{J_{ph}^{*}\left(V\right)}{J_{D}\left(V\right)}\right)$$

Figure 1d illustrates the photocurrent density change as a function of light intensity for BHJ and PHJ OPDs. The BHJ‐OPD and PHJ‐OPD have an LDR of 79 dB and 65 dB, respectively. The disparity in LDR values between the two OPD types is due to device performance, dark current density [D.C., JD(V)], and EQE. The D.C. of BHJ‐OPD is 1.54E‐8 mA cm^−2^, which is less than that of PHJ‐OPD, which is 3.83E‐8 mA cm^−2^. This is because the well‐mixed BHJ structure has an advantage in suppressing defect generation when compared to PHJ.^[^ ^9^ ^]^ Additionally, the photocurrent density [J*_ph_(*V*)] of BHJ‐OPD is greater than that of PHJ‐OPD when the applied voltage is fixed at −4 V. According to the analysis results above, the OPD devices developed here are sufficient to serve as VLC receivers.

### 2.2 OPD Frequency Response and Bandwidth

**Figure 2:**
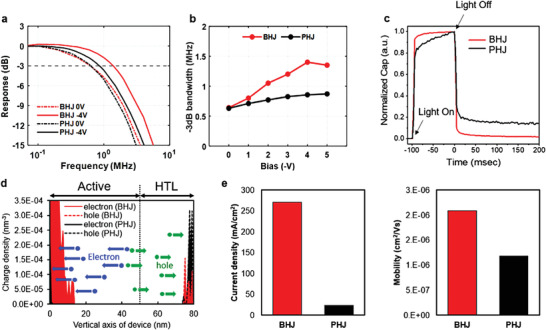
OPD frequency and dynamic characteristics. a) OPD frequency responses. b) −3 dB bandwidth by applied bias (0 V to −5 V). c) Transient capacitance data under light pulse (100 ms width). d) Charge distributions along with the vertical axis of the electrode interface obtained by kMC simulation and schematic illustrations of charge transport in the organic layers. e) Current density and charge mobility obtained by the kMC simulation.

To perform the function of a high‐speed OWC receiver, OPDs must exhibit a rapid response to changes in the intensity of incident light. The frequency responses of the tested BHJ and PHJ OPDs at 0 V and −4 V are shown in **Figure** **2**a. At 0 V reverse bias, or without external bias, the PHJ‐OPD and BHJ‐OPD exhibit comparable −3 dB bandwidths (*f*_3dB_). However, at −4 V bias, the −3 dB frequency of the BHJ‐OPD is increased to 1.4 MHz, while the frequency of the PHJ‐OPD is increased marginally by 0.85 kHz. Figure 2b shows the −3 dB bandwidth variation according to the applied voltages from 0 V to −5 V. The −3 dB bandwidth of the BHJ‐OPD device noticeably increases until −4 V, starting at 0.65 MHz (at zero bias) and reaching a maximum of 1.4 MHz (at −4 V bias). When −5 V bias is used, the bandwidth marginally decreases to 1.35 MHz. By comparison, the PHJ‐OPD gradually increases the −3 dB bandwidth up to 0.85 MHz at the bias of −5 V.

Numerous analysis techniques were used to determine the dynamic characteristics of OPD devices, including transient capacitance measurement,^[^ ^38^ ^]^ time‐dependent photo‐current measurement, and time‐delayed collection field measurement (TDCF).^[^ ^39^, ^40^ ^]^ The following equation can be used to determine the bandwidth of OPD.^[^ ^41^ ^]^

(3) $$-3\;\mathrm{dB}=20\cdot \log\frac{i\left(f_{3dB}\right)}{i_{0}}$$

where *i*_0_ and *i*(*f*_3dB_) are photocurrent intensity at steady‐state and −3 dB bandwidth, respectively.

OPD devices were optimized to maximize the −3 dB bandwidth when used as a receiver for rapidly changing optical signals. According to Equation 4, the total cutoff frequency can be calculated from the RC limited cutoff frequency (*f_RC_*) and transit limited cut‐off frequency (*f_tr_*).^[^ ^38^ ^]^

(4) $$\frac{1}{f_{3dB}^{2}}=\frac{1}{f_{RC}^{2}}+\frac{1}{f_{tr}^{2}}$$

RC limited time constant *t_RC_* could be derived from total series resistance (*R_s_*) and capacitance of OPD (C) as shown in Equation 5.^[^ ^42^ ^]^ The OPD devices were optimized to maximize the *f_RC_* and *f_tr_* and the device properties were analyzed.

(5) $$t_{RC}=\frac{0.35}{f_{RC}}=R_{s}\;C=R\varepsilon \frac{A}{d}$$

where *ε*  is the permittivity of dielectric material. To develop high‐speed OPD for optical communication, the active device area (*A*) and the thickness of the OPD (d) must be considered. As stated previously, we used a pair of reflecting electrodes to reduce the thickness of the OPD device while maintaining a 2 mm × 2 mm single pixel area to maximize photo‐response. To characterize the dynamic property of capacitance change in an OPD receiver under illumination with a 553 nm light pulse (pulse width = 100 ms, light intensity = 1 mW cm^−2^, and applied bias = −4 V), the transient capacitance was measured. As illustrated in Figure 2c, we measured and compared the normalized capacitance changes of BHJ‐OPD and PHJ‐OPD devices.^[^ ^43^ ^]^ Capacitance response to light illumination was significantly faster for BHJ‐OPD (*t*_rise,90%_ = 6.3 µsec) than for PHJ‐OPD (*t*_rise,90%_ = 65.3 µsec) and rapidly stabilized to steady‐state after light removal. After a single pulsed light illumination, the capacitance of the PHJ‐OPD could not reach the baseline, indicating that any remaining charges in the OPD cannot be fully discharged during the repeated charge‐discharge step. The results of the transient capacitance analysis support the −3 dB bandwidth measurement of the OPD (Figure 2a,b).

Additionally, we used kinetic Monte Carlo (kMC) simulation to confirm the charge transport mechanism for both types of OPD. Charge transport was calculated using the kMC simulation and the charge parameters were determined using DFT calculations for atomistic morphologies.^[^ ^44^, ^45^, ^46^, ^47^ ^]^ The transport of charge carriers (electron and hole) is illustrated in Figure 2d, along with the vertical axis of the electrode interface, as determined by the kMC simulation using an initial charge of a hundred electrons and holes randomly placed in the organic layers. Charges were excluded from the organic layers into the electrode without charge accumulation at the organic layer interfaces, although some electrons were collected at the electrode interface in the case of the BHJ. The current density and mobility of charge carriers on the BHJ and PHJ‐OPD are shown in Figure 2e. The charge mobility of the BHJ‐OPD is greater than that of the PHJ, which implies that the BHJ has greater charge mobility than the PHJ. Both experimental and theoretical studies demonstrate unequivocally that the BHJ provides greater charge mobility and superior photocurrent responses than the PHJ. Photo‐responsivity of OPD devices was characterized and summarized in Supplementary Note2. According to response measurements and analysis of recombination characteristics, the reason for better frequency response performance of BHJ‐OPD was confirmed experimentally.

### 2.3 BHJ‐OPD‐Based Receiver and Communication System

**Figure 3:**
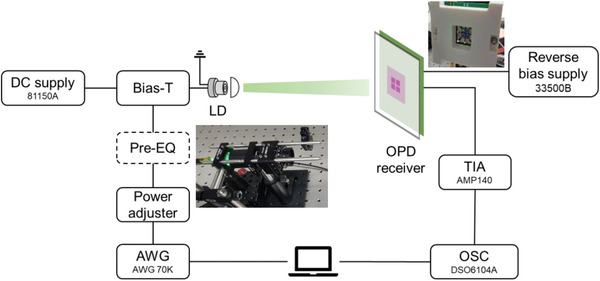
Communication performance test system setup. The system is composed of communication equipment, LD based transmitter module, OPD receiver module, signal conditioning, and reception parts.

**Figure** **3** depicts the BHJ‐OPD communication system in its initial configuration, as well as images of the laser diode (LD) and OPD modules. First, an arbitrary waveform generator (AWG) generates an analog signal that is sent to the LD module via a bias‐T unit with a direct current (DC) bias. To control the optical power and beam size, the green light from the LD module is passed through optics and optomechanical components such as a lens, an aperture, and a polarizer. For the stable experiment, the OPD holder and PCB are designed and printed in the manner shown in the picture inset of the OPD module. It is investigated at a 0.3 m link distance.

The OPD is biased using a reverse voltage generated by the DC function of a waveform generator for an automatic measurement system. The reverse bias can be used to increase the bandwidth and responsivity of the OPD. We primarily verified the zero bias and −4 V bias cases in this work. The former enables practically appealing applications that do not require external bias, while the latter enables higher performance with the highest measured bandwidth. The converted current signal is then transformed into a voltage signal by a transimpedance amplifier (TIA). The voltage signal is then captured in the analog domain, sampled, and quantized for analog to digital conversion (ADC) using an oscilloscope (OSC). The received signal is then processed in a computer.

**Figure 4:**
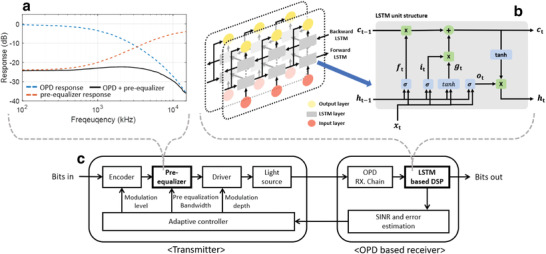
Flow of signal processing. a) Frequency responses of the applied preequalizer. b) Structure of the applied LSTM‐based DSP module. c) Adaptive control and feedback model including both preequalization and LSTM‐based DSP modules.

A preequalizer can be used to compensate for the innate low bandwidth of OPDs. As illustrated in **Figure** **4**a, the system bandwidth can be increased by using a preequalizer. When a circuit designed as an inverse of OPD's frequency response is applied, the flat part of the response can be further extended toward high‐frequency components. It means that the 3 dB bandwidth of the receiver is enhanced, leading to an improved communication performance. However, the response of the low‐frequency component has decreased as well, resulting in a signal‐to‐noise ratio (SNR) penalty. Thus, when designing the pre‐equalizer, a careful balance of bandwidth and available optical power should be maintained.

We employ a long short‐term memory (LSTM) network as a sequential data processor.^[^ ^48^ ^]^ Multiple information gates are contained within a single LSTM unit. Essentially, a forget gate determines whether to preserve the information, and then an input gate determines which information should be updated. The output is calculated and sent to the next layer using the results of information gates. All steps in this process are influenced by the previous results. Thus, the LSTM method can be used to treat a sequential data set containing severe ISI, such as a communication signal, in order to predict the original data and mitigate the problem. Bidirectional LSTMs (bi‐LSTMs), in particular, which are structured with forward and backward LSTMs, can learn more efficiently.

### 2.4 Communication Performances

**Figure 5:**
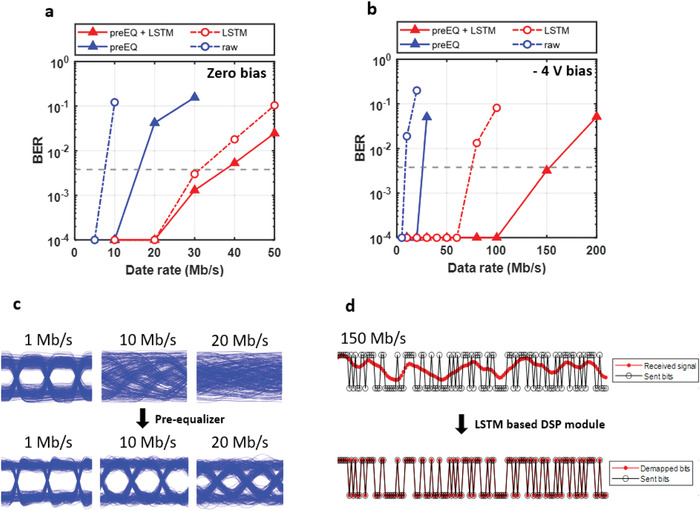
Communication performances. a) BER graph applying zero reverse bias on OPD. b) BER graph applying a reverse bias of 4 V on OPD. Both graphs show comparison results with and without pre‐EQ, and with and without LSTM‐based DSP. c) The eye‐diagrams at 1 Mbps, 10 Mbps, and 20 Mbps with and without preequalizer (at −4 V bias). d) Results of LSTM‐based DSP module at 150 Mbps, with a reverse bias of 4 V on OPD and preequalization used.

The bit‐error‐rate (BER) graphs of the cases with zero bias on the OPD are shown in **Figure** **5**a. When no compensation scheme is used (indicated by “raw”), only 5 Mbps is achieved within the forward error correction (FEC) threshold ( = 3.8 × 10^−3^ BER). However, when a preequalizer is used in conjunction with an LSTM‐based DSP, 30 Mbps (x8 of the previous best) is achieved while remaining within the FEC threshold.

Figure 5b shows the BER curves with an OPD reverse bias of 4 V. Overall, data rates are increased as a result of the increased responsivity and bandwidth. Using only the preequalizer, 20 Mbps is still achievable. Using a preequalizer and LSTM‐based DSP, it is possible to achieve 150 Mbps below the BER threshold. This rate is 40 times faster than the best results published previously.^[^ ^20^ ^]^

The three different first‐order preequalizers are compared in this work based on their response shapes and 3 dB bandwidth. The characteristics of each preequalizer are summarized in Note S2 of the Supporting Information. The eye diagrams at 1 Mbps, 10 Mbps, and 20 Mbps with and without the preequalizer are shown in Figure 5c. In all cases, a reverse bias of 4 V is applied. As illustrated in the figure, the preequalizer enabled clear eye‐opening in all three cases by compensating for the OPD's frequency response with the inverse transfer function. Without the preequalizer, the clear eye‐opening is monitored at a maximum of 1 Mbps.

Figure 5d illustrates the performance of an LSTM‐based DSP module operating at 150 Mbps with a reverse bias of 4 V applied to the OPD. Significant inter‐symbol interferences are observed in the received signal (upper figure). This is primarily because the modulated rate is significantly greater than the preequalized bandwidth. Nonetheless, the LSTM‐based DSP module successfully de‐mapped the aliased signal (see lower figure), resulting in a BER below the FEC threshold.

The proposed method is compared to DCO‐OFDM^[^ ^49^ ^]^ in terms of performance. For DCO‐OFDM, a bit and power loading scheme is used which allocates the appropriate bits and power to each subcarrier based on the channel condition being measured. Additional operational details and associated results for the adaptive DCO‐OFDM can be found in Supporting Information (Note S3).

**Figure 6:**
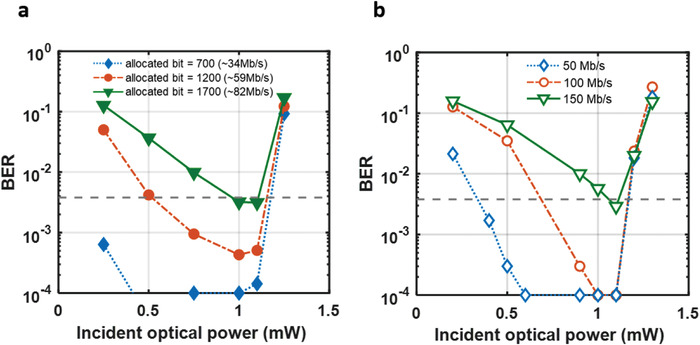
Communication performances. BER graph applying various optical power on OPD. a) adaptive DCO‐OFDM with 700, 1200, and 1700 allocated bits. b) bi‐LSTM DSP based 2‐PAM at 50 Mbps, 100 Mbps, 150 Mbps.

**Figure** **6**a and Figure 6b illustrate the BER graphs for the DCO‐OFDM and bi‐LSTM DSP‐based 2‐PAM, respectively, when various optical powers are applied to the OPD at various data rates. By increasing the optical power, the BER decreases for all rates. The performance of DCO‐OFDM with 700, 1200, and 1700 allocated bits is shown in Figure 6a. Each allocated bit corresponds to the following data transfer rates: 34 Mbps, 59 Mbps, and 82 Mbps. In this case, the fastest rate of ≈82 Mbps is possible within the FEC threshold. In comparison, the proposed scheme based on a 2‐PAM signal achieves a data rate of 150 Mbps with a BER of 3.05 × 10^−3^ as shown in Figure 6b. However, an abrupt increase in BER is observed beyond the incident optical power of 1.1 mW. This is primarily because the TIA has a limited linear dynamic range with hard clipping. Hence, the optical link budget is carefully designed and implemented, complete with a power back‐off plan.

## 3 Conclusion

**Figure 7:**
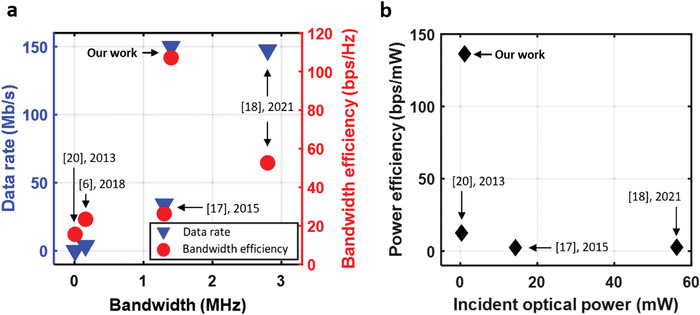
Comparisons with previous works. a) Data rate and bandwidth efficiency compared with previous works. b) Data‐rate corresponding to incident optical power compared with previous works.

We described the development of an OPD receiver for high‐speed optical wireless communication. Two different types of photoelectric conversion layers (bulk heterojunction and planar heterojunction) were analyzed and optimized. The BHJ‐OPD used in this work showed a bandwidth of 1.4 MHz at a −3 dB gain, which is estimated to RC limited cutoff frequency under the applied conditions.^[^ ^50^ ^]^ When the proposed communication schemes were implemented, a single channel system achieved a data rate of 150 Mbps. This is the fastest data rate achieved for the OWC demonstration using a VLC system based on OPD. Additionally, as illustrated in **Figure** **7**a, the result of this work is remarkably efficient in terms of bandwidth efficiency (the maximum data rate that can be transmitted over a given bandwidth) when compared to the previous research.^[^ ^6^, ^17^, ^18^, ^20^ ^]^ Furthermore, as illustrated in Figure 7b, the optical power required to achieve the fastest rate reported in this work is significantly less than the power levels required for previous results.

## 4 Experimental Section

### OPD Fabrication

The selenophene linked donor material (PSe) was synthesized in the laboratory.^[^ ^32^ ^]^ C60 as the acceptor material was purchased from Frontier Carbon Coup. The OPDs were fabricated using a patterned ITO/Ag/ITO film (Intree, TC‐S450). A 30‐nm‐thick HTL layer was deposited on the anode using vacuum thermal evaporation at a deposition rate of 0.01 nm s^−1^ and a base pressure of < 2 × 10^−7^ Pa. The photoelectric conversion layer was then deposited at a thickness of 50 nm. Finally, the 15‐nm‐thick Ag cathode was evaporated using vacuum thermal deposition with the aid of a shadow mask. Following cathode deposition, glass encapsulation using an ultraviolet (UV) curable resin as a sealant was immediately performed under a nitrogen atmosphere.

### OPD Characterization

The current density‐voltage characteristics were determined using a Keithley K4200 semiconductor parameter analyzer, and the EQE was determined using a spectral incident photon‐to‐electron conversion efficiency measurement system that generates monochromatic light via an optical filter from an ozone‐free Xe lamp operating at a chopper frequency of 50 Hz. The incident monochromatic light intensity was determined using a calibrated Si‐PD (Hamamatsu S1337). The measured intensity was 176 µW cm^−2^ at a wavelength of 540 nm.

To determine the frequency response of the photo‐current a 500 MHz bandwidth oscilloscope (Le Croy Wavejet 352A), an AFG function generator (GW instek), and a green laser diode (*λ* = 553 nm, 10 mW cm^−2^) were used.

The transient photocurrent of OPDs was determined using pulsed laser illumination from a Continuum Surelite SL II‐10 (peak wavelength: 545 nm and pulse width: 6 ns). The potential bias was ‐3 V applied to the OPDs, and the photo‐current was converted to voltage using a DLPCA100 current amplifier, from which the voltage output was measured using a Keysight DSO9104A digital storage oscilloscope.

### kMC Simulation

The kMC simulations were conducted using Nanomatch GmbH's multiscale modeling package. The parameters describing the charge transport properties between organic molecules in this kMC simulation were determined using density functional theory (DFT) calculations on molecular pairs with atomistic morphologies, as illustrated in Figure 1a.^[^ ^40^, ^44^ ^]^ Atomistic morphologies were generated using the Deposit module included in the package, which can generate disordered morphologies resembling physical vapor deposition.^[^ ^45^ ^]^ Molecular pairs were extracted for DFT calculations using a cutoff distance of 4 Å in nearest neighbor distances. The Marcus theory was used to calculate the charge hopping rate between molecules using the reorganization energy, transfer integrals, and energetic disorder parameters obtained from DFT calculations.^[^ ^46^ ^]^ The charge transport in the stacked organic layers was simulated using the kMC simulation with a charge hopping rate of ≈1 µsec. The organic layers were randomly implanted with hundreds of electrons and holes, respectively, and the electrons and holes were then transported to electrodes. Current density and charge mobility were calculated using charge carrier trajectory data obtained from the kMC simulation. kMC simulations were ran ten times for each device and calculated the average values of the density profile, current density, and mobility of charge carriers with various initial electron and hole arrangements.

### Communication Methods

MATLAB was used to generate and control the DCO‐OFDM and PAM signals using an AWG (Tektronix, AWG70000). Due to the output voltage limitation of AWG, the power adjusters such as an amplifier (Mini‐Circuits, ZPUL‐30P+) and an attenuator were used. Additionally, these signals were supplemented with a DC component via a bias‐T (Mini‐Circuits, ZFBT‐6GW+) and a DC function of an AWG (Keysight, 81150A) for an automatic measurement system. The LD (Thorlabs, PL520) was then operated at its center wavelength of 520 nm. To control the beam size, the green light from the LD was passed through optics and optomechanical components such as a lens and an aperture. A polarizer was placed between the transmitter and receiver to allow for precise power adjustment. On the receiver side, the OPD was encapsulated in a 3D‐modeled holder and PCB for the stable experiment. Tx and Rx were separated by 50 cm. The OPD is biased by applying a reverse voltage generated from a DC function of an AWG (Keysight, 33500B). The reverse bias range for OPD was primarily applied between 0 V and 5 V. The OPD detected the light signal and converted it to a current signal. Then, using a trnasimpedence amplifier (Thorlabs, AMP130), the current signal was converted to a voltage signal. The voltage signal was chaptered using an oscilloscope (Keysight, MSO 6104A), and the digital signal processing (DSP) was performed using MATLAB. Due to the limitation of TIA input power, an optical power meter (Thorlabs, PM400) was used to determine the received optical power.

## Conflict of Interest

The authors declare no conflict of interest.

## Supporting information

Supporting Information

## Data Availability

The data that support the findings of this study are available from the corresponding author upon reasonable request.

## References

[1] M. Z. Chowdhury , M. Shahjalal , M. K. Hasan , Y. M. Jang , Appl. Sci. 2019, 20, 4367.

[2] A. Mathew , in Proceedings of International Conference on Sustainable Expert Systems, 2021, 3, 176.

[3] M. Z. Chowdhury , M. K. Hasan , M. Shahjalal , E. B. Shin , Y. M. Jang , International Conference on Artificial Intelligence in Information and Communication (ICAIIC), Institute of Electrical and Electronics Engineers Inc., Okinawa, Japan 2019.

[4] S. R. Teli , S. Zvanovec , Z. Ghassemlooy , IEEE Int. Conf. on Internet of Things and Intelligence System (IOTAIS), Institute of Electrical and Electronics Engineers Inc., Bali, Indonesia 2018.

[5] S. A. H. Mohsan , A. Mazinani , W. Malik , I. Younas , N. Q. H. Othman , H. Amjad , A. Mahmood , Int. J. Adv. Comput. Sci. Appl. 2020, 11, 14.

[6] C. W. Chow , H. Y. Wang , C. H. Chen , H. W. Zan , C. H. Yeh , H. F. Meng , IEEE Access 2018, 6, 7625.

[7] S. Cho , H. Chun , Opt. Express 2021, 29, 28901. 34615010 10.1364/OE.434880

[8] S. Limbu , K. B. Park , J. Wu , H. Cha , S. Yun , S. J. Lim , H. Yan , J. Luke , G. Ryu , C. J. Heo , S. Kim , Y. W. , Jin , J. Durrant , J. S. Kim , ACS Nano 2021, 15, 1217. 33332092 10.1021/acsnano.0c08287

[9] N. Suganuma , C. J. Heo , D. Minami , S. Yun , S. Park , Y. Lim , F. Fang , B. Choi , K. B. Park , Adv. Electron. Mater. 2021, 8, 2100539.

[10] S. Heo , J. Lee , G. H. Lee , C. J. Heo , S. H. Kim , D. J. Yun , J. B. Park , K. Kim , Y. Kim , D. Lee , G. S. Park , H. Y. Cho , T. Shin , S. Y. Yun , S. Kim , Y. W. Jin , K. B. Park , Sci. Rep. 2020, 10, 219. 31937814 10.1038/s41598-019-57087-2 PMC6959276

[11] C. Vega‐Colado , B. Arredondo , J. C. Torres , E. López‐Fraguas , R. Vergaz , D. Martín‐Martín , J. M. Sánchez‐Pena , Sensors 2018, 18, 3045. 10.3390/s18093045 PMC6165323 30213031

[12] A. Basir , H. Alzahrani , K. Sulaiman , F. Muhammadsharif , S. M. Abdullah , A. Mahmoud , R. Bahabry , M. Alsoufi , T. Bawazeer , S. F. A. Sani , Mater. Sci. Semicond. Process. 2021, 131, 105886.

[13] G. Simone , M. Dyson , S. J. Meskers , R. J. Janssen , G. Gelinck , Adv. Funct. Mater. 2020, 30, 1904205.

[14] N. Strobel , N. Droseros , W. Kontges , M. Seiberlich , M. Pietsch , S. Schlisske , F. Lindheimer , R. Schroder , U. Lemmer , M. Pfannmoller , N. Banerji , G. Hernandez‐Sosa , Adv. Mater. 2020, 32, 1908258. 10.1002/adma.201908258 32068919

[15] B. Arredondo , B. Romero , J. M. S. Pena , A. Fernández‐Pacheco , E. Alonso , R. Vergaz , C. D. Dios , Sensors 2013, 13, 12266. 24036584 10.3390/s130912266 PMC3821371

[16] H. S. Ryu , S. Y. Park , T. H. Lee , J. Y. Kim , H. Y. Woo , Nanoscale 2020, 12, 5792. 32129404 10.1039/d0nr00816h

[17] S. Zhang , D. Tsonev , S. Videv , S. Ghosh , G. Turnbull , I. D. W. Samuel , H. Haas , Optica 2015, 2, 607.

[18] I. Tavakkolnia , L. Jagadamma , R. Bian , P. Manousiadis , S. Videv , G. Turnbull , I. D. W. Samuel , H. Haas , Light Sci. Appl. 2021, 10, 41. 33623027 10.1038/s41377-021-00487-9 PMC7902835

[19] I. K. Kim , B. N. Pal , M. Ullah , P. L. Burn , S. C. Lo , P. Meredith , E. B. Namdas , Adv. Opt. Mater. 2015, 3, 50.

[20] Z. Ghassemlooy , P. A. Haigh , F. Arca , S. F. Tedde , O. Hayden , I. Papakonstantinou , S. Rajbhandari , Photonics Res. 2013, 1, 65.

[21] P. A. Haigh , Z. Ghassemlooy , I. Papakonstantinou , F. Arca , S. F. Tedde , O. Hayden , E. Leitgeb , IEEE. Photonics Technol. Lett. 2014, 26, 1295.

[22] A. C. Alexandru , G. M. Sandulescu , M. Bistran , 2020 IEEE Int. Conf. on Automation, Quality and Testing, Robotics (AQTR), IEEE, Atlanta, GA, USA 2020.

[23] Y. Wang , J. Kublitski , S. Xing , F. Dollinger , D. Spoltore , J. Benduhn , K. Leo , Mater. Horiz. 2022, 9, 220. 34704585 10.1039/d1mh01215k

[24] Z. Lan , Y. S. Lau , Y. Wang , Z. Xiao , L. Ding , D. Luo , F. Zhu , Adv. Opt. Mater. 2020, 8, 2001388.

[25] S. R. Forrest , Nature 2004, 428, 911. 15118718 10.1038/nature02498

[26] T. Sekitani , T. Someya , Adv. Mater. 2010, 22, 2228. 20229571 10.1002/adma.200904054

[27] C. W. Tang , Appl. Phys. Lett. 1986, 48, 183.

[28] K. J. Baeg , M. Binda , D. Natali , M. Caironi , Y. Y. Noh , Adv. Mater. 2013, 25, 4267. 23483718 10.1002/adma.201204979

[29] R. D. Jansen‐van Vuuren , A. Armin , A. K. Pandey , P. L. Burn , P. Meredith , Adv. Mater. 2016, 28, 4766. 27111541 10.1002/adma.201505405

[30] C. Labanti , J. Wu , J. Shin , S. Limbu , S. Yun , F. Fang , S. Y. Park , C.‐J. Heo , Y. Lim , T. Choi , H.‐J. Kim , H. Hong , B. Choi , K.‐B. Park , J. R. Durrant , J.‐S. Kim , Nat. Commun. 2022, 13, 3745. 35768429 10.1038/s41467-022-31367-4 PMC9243077

[31] G. Yu , J. Gao , J. C. Hummelen , F. Wudl , A. J. Heeger , Science 1995, 270, 1789.

[32] Y. Lim , S. Yun , D. Minami , T. Choi , H. Choi , J. Shin , S. Kim , ACS Appl. Mater. Interfaces 2020, 12, 51688. 33164496 10.1021/acsami.0c14237

[33] C. J. Heo , T. Motoyama , G. H. Lee , S. Yun , S. Park , Y. Lim , K. B. Park , Org. Electron. 2021, 106154.

[34] J. M. Lupton , R. Koeppe , J. G. Müller , J. Feldmann , U. Scherf , U. Lemmer , Adv. Mater. 2003, 15, 1471.

[35] S. Shafian , K. Kim , ACS Appl. Mater. Interfaces 2020, 12, 53012. 33172259 10.1021/acsami.0c17183

[36] L. Yang , D. Guo , J. Li , G. He , D. Yang , A. Vadim , D. Ma , Adv. Funct. Mater. 2022, 32, 2108839.

[37] M. Kielar , O. Dhez , G. Pecastaings , A. Curutchet , L. Hirsch , Sci. Rep. 2016, 6, 39201. 28004819 10.1038/srep39201 PMC5177896

[38] S. Demirezen , S. A. Altındal Yerişkin , Polym. Bull. 2020, 77, 49.

[39] A. Tataroglu , R. Ocaya , A. Dere , O. Dayan , Z. Serbetci , A. G. Al‐Sehemi , F. J. Yakuphanoglu , Electron. Mater. 2018, 47, 828.

[40] A. Paulke , S. D. Stranks , J. Kniepert , J. Kurpiers , C. M. Wolff , N. Schön , D. Neher , Appl. Phys. Lett. 2016, 108, 113505.

[41] N. Strobel , M. Seiberlich , R. Eckstein , U. Lemmer , G. Hernandez‐Sosa , Flexible Printed Electron. 2019, 4, 043001.

[42] K. A. Kato , S. Hata , J. Yoshida , A. Kozen , In 4th International Conference on Indium Phosphide and Related Materials, Institute of Electrical and Electronics Engineers Inc., Newport USA 1992, 254.

[43] H. Shekhar , A. Fenigstein , T. Leitner , B. Lavi , D. Veinger , N. Tessler , Sci. Rep. 2020, 10, 7594. 32372047 10.1038/s41598-020-64565-5 PMC7200686

[44] P. Friederich , F. Symalla , V. Meded , T. Neumann , W. Wenzel , J. Chem. Theory Comput. 2014, 10, 3720. 26588517 10.1021/ct500418f

[45] P. Friederich , V. Meded , F. Symalla , M. Elstner , W. Wenzel , J. Chem. Theory Comput. 2015, 11, 560. 26580913 10.1021/ct501023n

[46] T. Neumann , D. Danilov , C. Lennartz , W. Wenzel , J. Comput. Chem. 2013, 34, 2716. 24114652 10.1002/jcc.23445

[47] F. Symalla , S. Heidrich , P. Friederich , T. Strunk , T. Neumann , D. Minami , Adv. Theory Simul. 2020, 3, 1900222.

[48] X. Dai , X. Li , M. Luo , Q. You , S. Yu , Appl. Opt. 2019, 58, 6079. 31503928 10.1364/AO.58.006079

[49] H. Chun , S. Rajbhandari , D. Tsonev , G. Faulkner , H. Haas , D. O'Brien , in 2015 IEEE International Conference on Communication Workshop (ICCW), IEEE, London, UK 2015, 1392.

[50] S. Valouch , M. Nintz , S. W. Kettlitz , N. S. Christ , U. Lemmer , IEEE Photonics Technol. Lett. 2022, 24, 596.

