# Supplementary material for: Small Molecule Based Organic Photo Signal Receiver for High‐Speed Optical Wireless Communications
Source: Adv Sci (Weinh). 2022 Oct 3;9(32):2203715. doi: 10.1002/advs.202203715 (PMC9661864; doi:10.1002/advs.202203715)
Supplement: Supplementary file 1 — Supporting Information [file ADVS-9-2203715-s001.pdf]

Supporting Information for

Small Molecule Based Organic Photo Signal Receiver for High Speed Optical Wireless Communications

Seonghyeon Cho[#], Chul-Joon Heo[#], Younhee Lim, Seoyeon Oh, Daiki Minami, Minseok Yu, Hyunchae Chun, Sungyoung Yun, Hwijoung Seo, Feifei Fang, Jeong-Il Park, Cheol Ham, Jisoo Shin, Taejin Choi, Juhyung Lim, Hyeong-Ju Kim, Hye Rim Hong, Hiromasa Shibuya, Jeoungin Yi, Byoungki Choi, and Kyung-Bae Park**

Supplementary Note 1. Electrode effects on OPD devices

To clarify the effect of electrode on OPD performances, EQE and bandwidth of BHJ-OPD with cavity structure (50 nm BHJ) were compared with BHJ-OPD with transparent electrode pairs (100 nm BHJ). Although the total thickness of light-absorbing layer of cavity OPD was decreased to half of non-cavity OPD, EQE at 550nm was comparable, and -3dB bandwidth was improved from 37 kHz to 1.4 MHz under -4V operating condition.

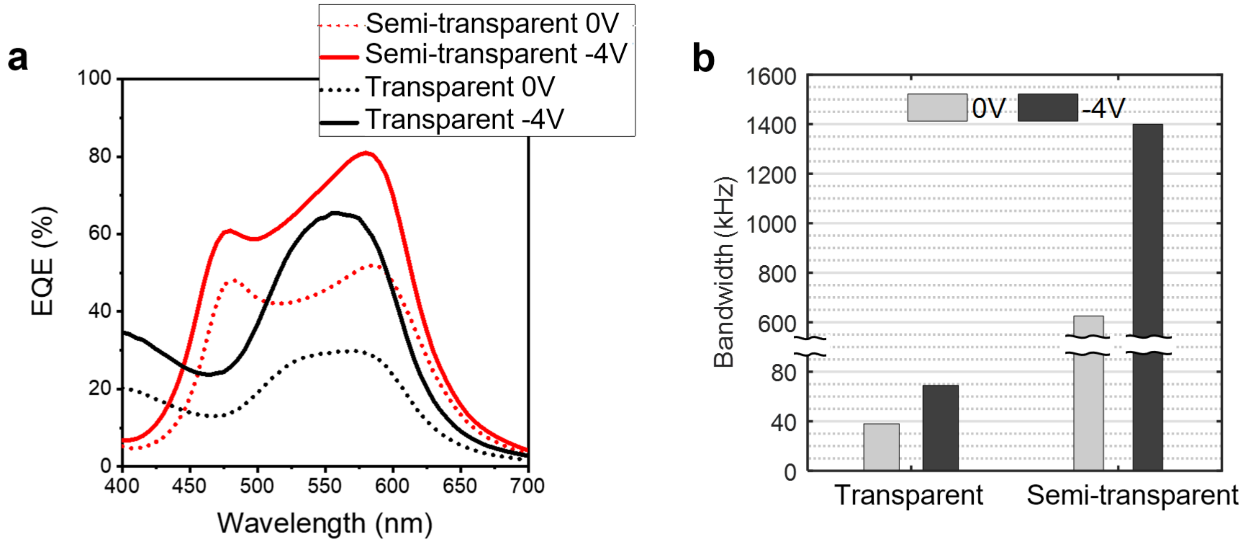

Figure S1. Electrode effect on OPD devices. a) EQE spectra of two different OPDs (OPD with semi-transparent electrode and with transparent electrode) under 0V, -4V operating conditions. b) -3dB bandwidth of OPD devices with semi-transparent electrode and with transparent electrode.

Supplementary Note 2. OPD response analysis

The transit-limited cut-off frequency associated with charge mobility is also critical for the -3dB bandwidth of OPDs. On OPD devices, two types of transient current measurements were performed under light illumination: photo-responsive current measurements and current measurements with ms and ns level light pulses. (Figure S2 and Table S1 in Supporting Information (Supplementary Note1) summarize the such information for OPDs). Transient photocurrent measurements of OPD devices under pulsed light illumination (light intensity = 1 mW/cm^2, wavelength, λ = 553 nm) at a frequency of 2 kHz are shown in Figure S2a. The rise-

time (τ_{rise}) and fall-time (τ_{fall}) of OPDs were determined by the time required to reach 90% of the maximum signal after light-on and 10% of the maximum signal after light-off, respectively. The response time of BHJ-OPD (τ_{rise} =3.2 μsec, τ_{fall} =3.3 μsec) was significantly faster than PHJ-OPD (τ_{rise} =13.7 μsec, τ_{fall} =12.7 μsec). Following an investigation of microsecond-level photo-response of OPD receiver, devices were constructed to determine their effective mobility and charge recombination properties using transient photocurrent measurements under laser illumination.[9,33] The TDCF photocurrent transients measured without delay time are depicted in Figure S2b. The sample was kept at a pre-bias of 0.5 V during excitation and at a collection voltage (V_{coll}) of -3 V prior to collection. The effective mobility μ_{TDCF} of the OPDs was calculated from t_0 using Equation 6. In comparison to the mobility of PHJ-OPD, which is 3.0E-5 cm^2/Vs, the mobility of BHJ-OPD is 3.6E-5 cm^2/Vs.

$$\mu = \frac{d^2}{V \cdot t_0} \tag{1}$$

, where d is the thickness of the organic layer.

To determine the source of the difference in mobility between two device types, we analyzed the charge recombination dynamics using time-delayed TDCF measurements.[34] After a time delay of 300 ns to 20000 ns, transient photocurrent was measured and parameters relating to recombination dynamics were determined for each device by fitting the differential extracted charge (ΔQ/Δt) plot to the collected charge amount (Q_{coll}).

$$\begin{aligned} Q_{tot}(t+\Delta t) &= Q_{coll}(t+\Delta t) + Q_{pre}(t+\Delta t) \\ &= Q_{tot}(t) - \frac{k_2}{eAd}\left[Q_{coll}^2(t) + 2Q_{bg}Q_{coll}(t)\right]\Delta t \end{aligned} \tag{2}$$

, where *e*, *A*, and *d* are the elementary charge, device area and active layer thickness, respectively. The recombination of photogenerated free charge with the background charge (Q_{bg}) and time-independent bimolecular recombination associated with (k_2) are two major recombination mechanisms that can affect the charge mobility of the system. Experimentally, the faster photoresponse of BHJ-OPD was compared to that of PHJ-OPD using TDCF measurements with and without delay. In PHJ-OPD, the majority of free charges are generated at the interface between the n- and p-type films, and they have a greater chance of dissipating during their journey to the electrodes via first-order recombination with the background charge. However,

because light can generate free charges through the BHJ layer, second-order recombination may be the primary recombination mechanism for BHJ-OPD. Although bimolecular recombination occurs throughout the OPD, BHJ-OPDs have a higher net charge mobility due to the suppression of background charge generation, and the average travel path for free charges is shorter in BHJ-OPD than in PHJ-OPD under a fixed electrical field. As previously stated, recombination coefficients derived from TDCF measurements with delay can adequately explain this phenomenon. The parameter for bimolecular recombination, k_2, was greater for BHJ-OPD (1.7E-10 cm^{-3}/s) than for PHJ-OPD (1.9E-11 cm^{-3}/s). Furthermore, the parameter relating to first-order recombination (Q_{bg}) of BHJ-OPD (1.0E-9 C) was less than that of PHJ-OPD (2.3E-9 C).

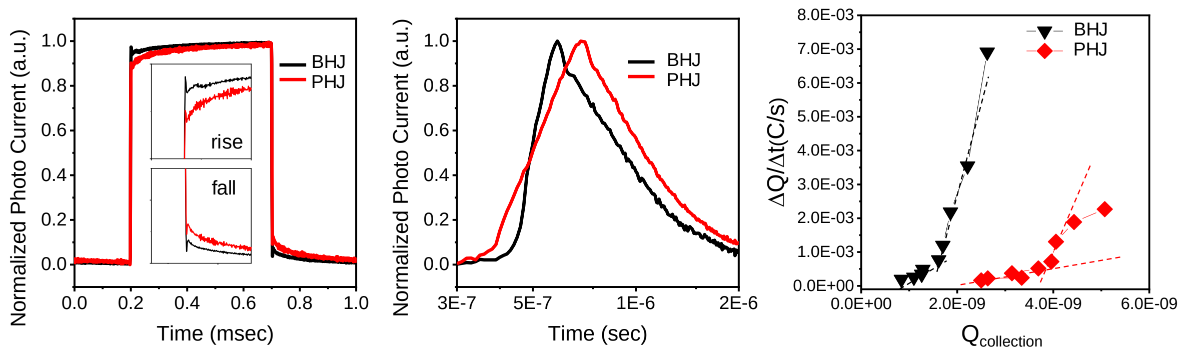

(a) (b) (c)

Figure S2. Response speed of OPD devices. a) Photo-response measurement of OPDs. (Insets: magnified current response during rise and fall, respectively) b) Transient photocurrent profiles obtained from TDCF measurement without delay time. c) Differential extracted charge ($\Delta Q/\Delta t$) vs. collected charge ($Q_{collection}$) for delays.

Table S1. Response properties of OPD receivers

Type	τ_{rise} [ms]	τ_{fall} [ms]	μ_{TDCF} [cm^2/Vs]	k_2 [cm^{-3}/s]	Q_{bg} [C]
(1) BHJ	3.2	3.3	3.6E-05	1.7E-10	1.0E-9
(2) PHJ	13.7	12.7	3.0E-05	1.9E-11	2.3E-9

Supplementary Note 3. Pre-equalizer circuit and response

The system's capability is limited by the organic photodiode's (OPD) -3dB bandwidth limitation of 1.4MHz at 4V reverse bias. Pre-equalization is frequently used to augment the visible light communication (VLC) system's limited bandwidth. On various order circuits, the analog pre-equalizer can be designed with a variety of shapes, slopes, and cut-off frequencies. In the current study, a simple first order pre-equalizer consisting of a resistor (R) and (C) a capacitor in parallel is used, as illustrated in Figure S3a. It is located between the arbitrary waveform generator and the light source, just before the transmitted signal lights are generated. Additionally, the modulated alternating current (AC) components from AWG are distorted to the required shape via a pre-equalizer.

Three circuits with varying shapes and -3dB frequencies are evaluated here by varying the values of R and C, designated as $\mathrm{APEQ1}(\mathrm{R}=510\Omega, \mathrm{C}=270\mathrm{pF})$, $\mathrm{APEQ2}(\mathrm{R}=820\Omega, \mathrm{C}=160\mathrm{pF})$ and APEQ3 $(\mathrm{R}=510\Omega, \mathrm{C}=270\mathrm{pF})$, respectively. The frequency responses of OPD and each APEQ are depicted in Figure S3b. The response of APEQ1 is flat, and the -3dB bandwidth is extended to ~6 MHz with an ~18dB attenuation of the DC response. APEQ 2 has a lower response gain (almost -3dB) but a wider -3dB bandwidth (~7 MHz) than APEQ 1. APEQ3 exhibits an arched response with a -3dB point of ~7 MHz. Figure S3c illustrates BER measurements and eye-diagram insets at a data rate of 30 Mbps after applying each APEQ. Due to the limited bandwidth, 30 Mbps with APEQ1 cannot meet the FEC threshold. Although the signal-to-noise ratio is relatively high in comparison to others, the eye opening is unclear, as illustrated in the inset (i). While APEQ2 and APEQ3 both reduce response, the BER satisfying the threshold is obtained due to the larger -3dB bandwidth. Notably, the APEQ2 eye diagram (inset (ii)) is more distinct than the APEQ one eye diagram (inset (iii)).

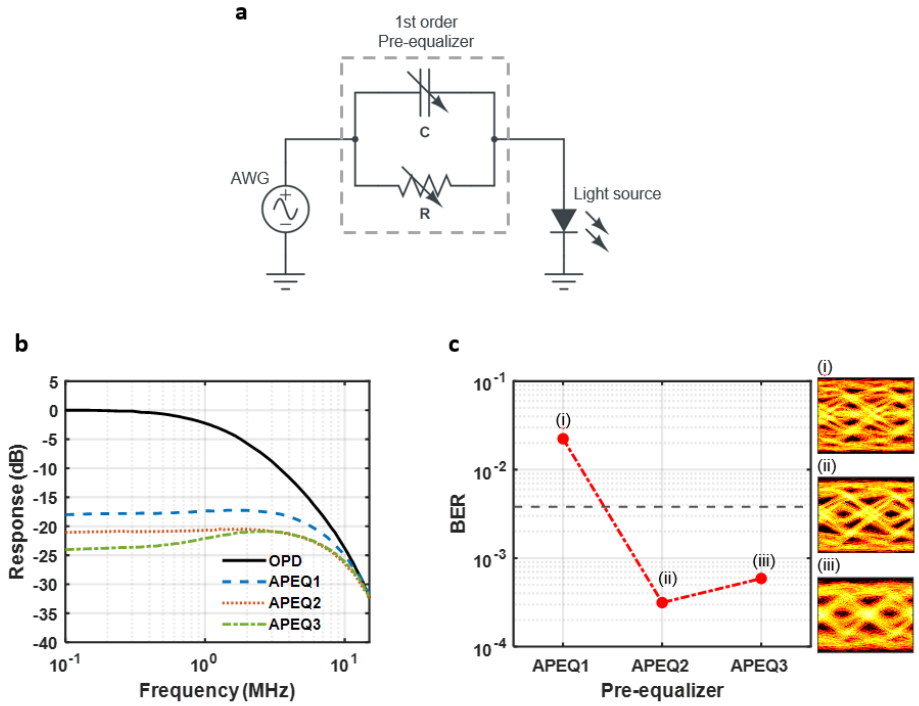

Figure S3. Analog pre-equalization analysis. a) transmittance circuit with the first-order pre-equalizer. b) OPD frequency response with and without the used analog pre-equalizers (APEQs). c) BER measurement with a data-rate of 30Mbps and eye-diagram at difference APEQ.

Supplementary Note 4. DCO-OFDM

Due to its high spectral efficiency, DC-biased optical orthogonal division multiplexing (DCO-OFDM) has been widely used to achieve the highest performance in optical wireless communication (OWC) systems. By applying DC bias to the optical OFDM signal in DCO-OFDM, the transmitted signal can be made unipolar, satisfying the requirements of both the intensity modulation (IM) and direct detection (DD) systems. Nonetheless, due to the high peak-to-average power ratio (PAPR), it is difficult to maintain a positive signal.[49] However, the clipping process ensures not only a positive signal but also the full dynamic range of the light source, resulting in a higher signal-to-noise ratio (SNR). The clipped OFDM signal $\mathrm{s_c}(n)$ can be expressed with unclipped OFDM signal $s_o(n)$:

$$s_c(n) = \alpha \times s_o(n) + N_c(n) \tag{3}$$

, where α is $\frac{\mathrm{E}[\mathrm{S}_c S_o]}{\sigma_{s_o}^2}$, and $N_c(n)$ denotes clipping noise. The signal is distorted by passing through the channel h(n) with the additive noise $N(n)$. The received signal r(n) can then be expressed as:

$$r(\mathrm{n}) = s_c(n) * h(n) + N_r(n) \tag{4}$$

, where $*$ indicates a convolution operation.

$$SNR(k) = \frac{\sigma_{s_o}^2 |H(k)|^2 \alpha^2}{\sigma_r^2(k) + \sigma_c^2(k)} = \frac{\alpha^2}{\frac{\sigma_r^2(k)}{\sigma_{s_o}^2 |H(k)|^2} + \frac{\sigma_c^2(k)}{\sigma_{s_o}^2 |H(k)|^2}} \tag{5}$$

$$= \frac{\alpha^2}{[SNR_r(k)]^{-1} + [SNR_c(k)]^{-1}} \tag{6}$$

Equations 5 and 6 express the SNR on each subcarrier in OFDM, where k denotes the sub-carrier index. It can be defined as $H(k)$, σ_r^2 and σ_c^2 , where $H(k)$ denotes the channel impact, σ_r^2 denotes the variance of the additive noise at the receiver, and σ_c^2 denotes the variance of the clipping noise, respectively, as shown in Equation (5). Then, this can be organized as shown in Equation (6) with available SNR_r and SNR_c meaning an effect of additive noise and clipping noise.

Using this available SNR on each subcarrier, the capacity (C) of the DCO-OFDM can be derived with the maximum number of bits (B_{max}) as:

$$C = \frac{B_{max}}{T} = \Delta f \sum_{k=1}^{\frac{N_{fft}}{2}-1} \{1 + SNR(k)\} \quad [\text{bits/sec}] \tag{7}$$

, where T is the time duration of a single DCO-OFDM frame. Δf is the distance between subcarriers and N_{fft} means the subcarrier size for the Fourier fast transform (FFT). To mitigate interference between OFDM symbols, the number of redundant subcarriers, N_{CP}, is added to the pure OFDM signal for the cyclic prefix (CP). The total subcarrier size N_{total} is expended in this case as $N_{fft} + N_{CP}$. When the following inequation is satisfied, $N_{fft} \gg N_{CP}$, $\frac{1}{T}$ converges to Δf. As a result, the B_{max} can be estimated by Equation 8.

$$B_{max} \cong \sum_{k=1}^{\frac{N_{fft}}{2}-1} \{1 + SNR(k)\} \quad \text{[bits]} \tag{8}$$

Particularly, the capacity of DCO-OFDM can be optimized by utilizing a channel adaptive bit and power loading scheme for high data rates. It determines the number of bits and the amount of power allocated to each subcarrier for M-level quadrature amplitude modulation (M-QAM) based on the channel condition. The level of QAM is simply calculated by $M_i = 2^{b_i}$ on i^{th} subcarrier. Although this method adds complexity to the system, it enables the DCO-OFDM to perform better than in the fixed case. Using this method, the data-rate can be calculated according to the channel condition as:

$$Data\,rate = \sum_{i=0}^{\frac{N_{fft}}{2}-1} \log_2 M_i \Big/ N_{total} \times \frac{f_{over}}{f_{clock}} \quad \text{[bits/sec]} \tag{7}$$

, where f_{over} and f_{clock} denote over sampling rate and system sampling clock, respectively.

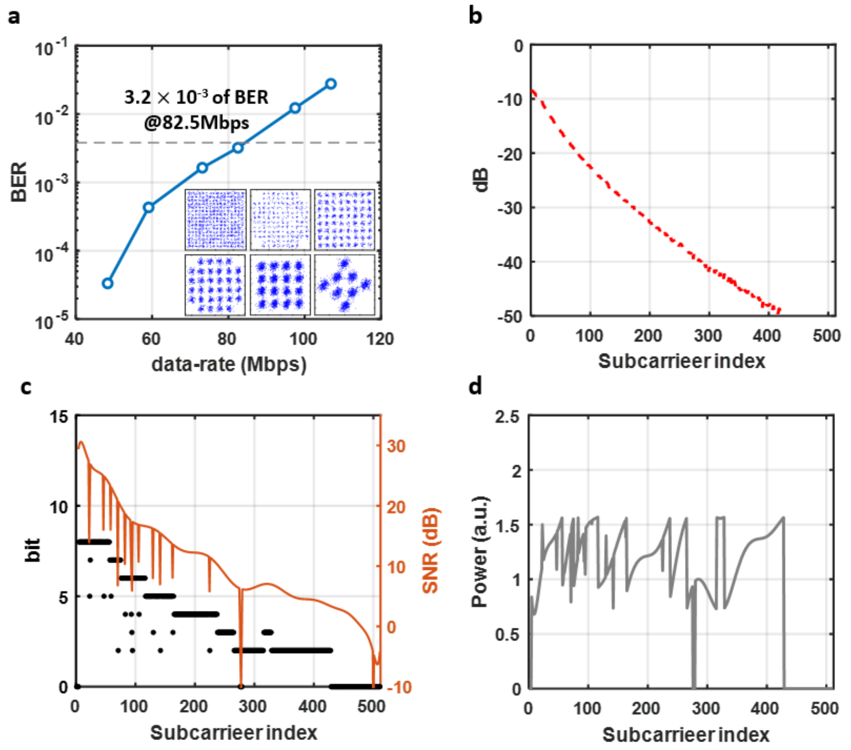

Figure S4. DCO-OFDM results. a) BER graph varying date-rate. b) estimated SNR. c) allocated bits. d) allocated energy.

Figure S4 illustrates the DCO-OFDM results of the experiment at an incident power of 1.1 mW. The Fourier Fast Transform (FFT) size and cyclic prefix (CP) length in this work are 1024 subcarriers and 10 length, respectively. Additionally, the optimal clipping size has been empirically determined to be three times the signal's standard deviation (3σ). The Hermitian generates the real signal using half of the subcarriers. Thus, except for the DC component, the final sub-carrier is 511. Additionally, it is located in the frequency domain at 25 MHz. Except for the pre-equalizer, the experimental setup and conditions are identical to those proposed in the paper for scheme-based experiments. In the case of DCO-OFDM, the pre-equalizer used in conjunction with M-level pulse amplitude modulation (M-PAM) reduces the SNR on each subcarrier rather than increasing it.

Figure S4a illustrates the measured bit-error-rate (BER) graph at various data rates. The achievable data rate is approximately 82.5 Mbps when the bit error rate (BER) is less than the

forward error correction (FEC) threshold ($= 3.8 \times 10^{-3}$). The figure also depicts the distinct constellations at an 82.5 Mbps data rate corresponding to M-QAM (M=8, 16, 32, 64, 128, 256)

Figure S4b, c and d illustrate the highest data-rate channel's estimated SNR, allocated bits and the optimized power loading scheme on each subcarrier, respectively.

Supplementary Note 5. Long Short-Term Memory neural network

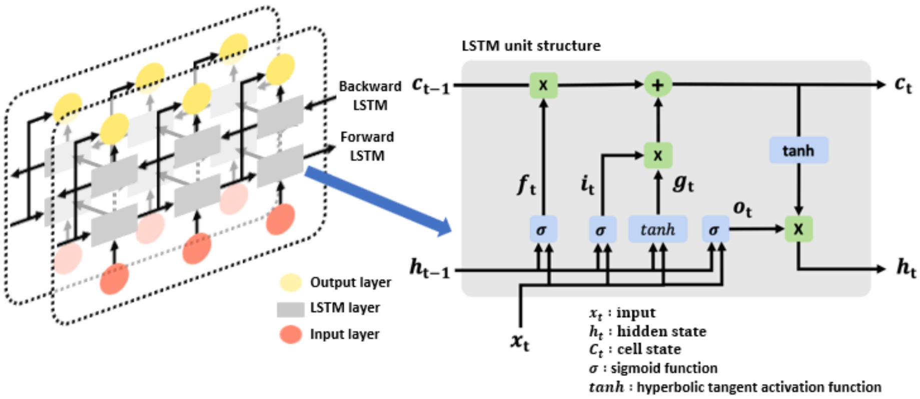

Figure S5. Diagram for neural network representing an LSTM unit with information gates

A long short-term memory (LSTM) network is a machine learning (ML) technique. It is a subset of the recurrent neural network (RNN) model that is capable of processing sequential data[40]. As illustrated in Figure S5, a single LSTM unit consists of an input gate (i), a forget gate (f), a cell candidate (g) and an output gate (o). Each gate has their own weights: input weight ($W_i\ W_f\ W_g\ W_o$), recurrent weight ($R_i\ R_f\ R_g\ R_o$) and layer bias ($b_i\ b_f\ b_g\ b_o$). Theses weights are updated depending on learning rate of each gate. Then, at the t step, the output of the input gate, forget gate, cell candidate, and output gate according to i_t, f_t, g_t, o_t are calculated as:

$$i_t = \sigma(W_i\, x_t + R_i h_{t-1} + b_i) \quad (10)$$

$$f_t = \sigma\left(W_f x_t + R_f h_{t-1} + b_f\right) \quad (11)$$

$$g_t = \tanh\left(W_g\, x_t + R_g h_{t-1} + b_g\right) \quad (12)$$

$$o_t = \sigma(W_o\, x_t + R_o h_{t-1} + b_o) \quad (13)$$

, where x_t is the current input. The cell state (c_t) and hidden state (h_t) are shared to the next LSTM unit to analyze the sequential relation of input data. These states on tth are calculated as:

$$c_t = f_t \odot c_{t-1} + i_t \odot g_t \tag{14}$$

$$h_t = o_t \odot \tanh(c_t) \tag{15}$$

, where $\odot$ indicates the element-wise multiplication of vectors and c_{t-1} is the previous cell state information. Initially, the forget gate determines whether or not to preserve information in the LSTM process. The input gate then determines the content to be updated in the cell. The cell candidate for the current cell state is determined. Following that, the cell state is updated using the results of the forget gate, input gate, and cell candidate, and the cell is passed to the following layer. Finally, the output gate calculates the output and sends it to the following layer as well. All steps in this process are influenced by the previous result of the output gate. Thus, the LSTM method can be used to treat a sequential data set containing severe ISI, such as a communication signal, in order to predict the original data and mitigate the problem. Bidirectional LSTMs (bi-LSTMs), in particular, which are structured with forward and backward LSTMs, can learn more efficiently.

Supplementary Note 6. Real-time demonstration

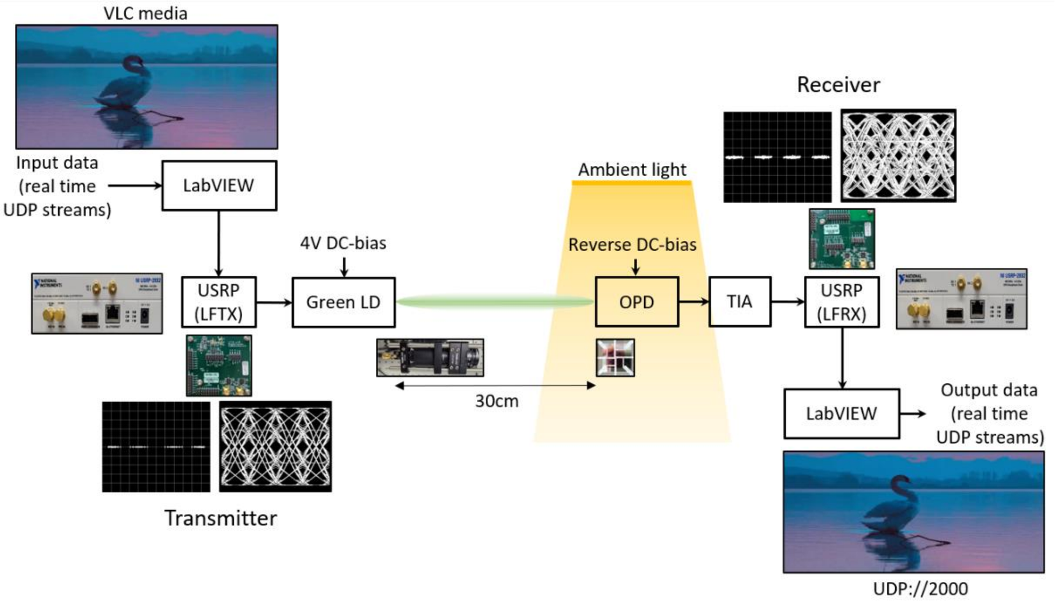

Figure S6. Real-time communication through UDP by using OPD receiver.

Figure S6 illustrates a real-time communication system utilizing an optical wireless communication system comprised of the green LD module, BHJ-OPD receiver, and USRP-LabVIEW-based real-time signal generator and sink systems. The real-time verification tests were conducted using LabVIEW, NI-LFTX, NI-LFRX, and NI-USRP-2932, all of which operate optimally at low frequencies between 0 and 30 MHz. The applied Modulation method and parameters for the experiment are four-level amplitude modulation (4-PAM), 4 samples per symbol, 800k sample rate, and 800k carrier frequency, respectively. The packetized stream using UDP protocol was transmitted through the communication channel. A typical ambient light level of 400 lux is applied for this demonstration. It is shown that through the applied 4-PAM signal, the video-streaming data is generated correctly and received. Although the eye diagrams show a reduction in eye opening at Rx, the received signal quality is sufficient to demodulate the received signal, resulting in no bit errors in this real-time communication via the user-datagram protocol (UDP).
